# Editorial: Inflammation and lipid signaling in disease pathogenesis

**DOI:** 10.3389/fphar.2025.1623707

**Published:** 2025-05-16

**Authors:** Paola Patrignani, Bernhard Brüne, Angelo Sala, Pietro Minuz, Dieter Steinhilber

**Affiliations:** ^1^ Department of Neuroscience Imaging and Clinical Science, and CAST, “G.d’Annunzio” University, School of Medicine, Chieti, Italy; ^2^ Department of Biochemistry I, Faculty of Medicine, Goethe University Frankfurt, Frankfurt, Germany; ^3^ Department of Pharmaceutical Sciences, University of Milan, Milan, Italy; ^4^ Department of Medicine, University of Verona, Verona, Italy; ^5^ Institute of Pharmaceutical Chemistry, Goethe University Frankfurt, Frankfurt, Germany

**Keywords:** inflammation, lipid mediators, eicosanoids, cyclooxygenase, leukotrienes, NSAIDs, inflammatory-related diseases

## Introduction

Inflammation is a highly coordinated biological response involving a network of immune and inflammatory cells, platelets, stromal and vascular components, and various molecular mediators (Ricciotti and FitzGerald, 2011; Patrignani and Patrono, 2015; Schebb et al., 2022; Yang et al., 2025). This complex process is essential for eliminating harmful stimuli, clearing damaged cells, and facilitating tissue repair. Although inflammation is crucial for host defense reactions, it can become maladaptive if it persists in chronic form or is excessively triggered, leading to pathophysiological states associated with various diseases, including malignancies, asthma, and cardiovascular conditions such as atherosclerosis and heart failure. Understanding the molecular and cellular pathways regulating inflammation is essential for developing targeted therapeutic strategies. In this Research Topic, studies have investigated novel mechanisms associated with inflammation, offering new insights into the intricate cellular dynamics involved. Furthermore, this Research Topic of studies addresses cutting-edge pharmacological approaches, highlighting promising therapeutic avenues to improve treatment efficacy.

Zhang et al. have investigated the role of interleukin-6 (IL-6) in the JAK2-STAT1/3 pathway during gout inflammation in patients with acute-phase gout (AG), intermittent gout (IG), and healthy controls. It was found that gout patients had lower mRNA levels of IL6, JAK2, and STAT1/3 than controls, indicating a negative feedback mechanism. In the AG group, IL-1β and IL6, JAK2 and STAT1/3 proteins increased significantly, while the IG group had elevated IL-1β but lower phosphorylated proteins. Higher IL-6 levels in AG may enhance JAK2 activation and inflammation. The study highlights the role of the IL-6/JAK2/STAT1/3 pathway in acute gout inflammation.

5-Lipoxygenase (5-LO), encoded by *ALOX5*, is involved in leukotriene biosynthesis, playing a key role in inflammatory diseases and linked to certain tumors. Hyprath et al. have investigated how the leukemogenic fusion protein MLL-AF4 upregulates *ALOX5* gene expression. It was demonstrated that MLL-AF4 and MLL-AF9 strongly activate the *ALOX5* promoter in B-lymphocytic cells, with MLL-AF4 effects mediated by the tandem GC box. Additionally, it was identified that several AF4 domains bind the super elongation complex and are essential for inducing *ALOX5* promoter activity.

Wickert et al. have discussed how ferroptosis influences inflammatory pathways and the effect of iron metabolism on immune cell ferroptosis during inflammation. Ferroptosis is an iron-dependent form of cell death characterized by lipid peroxidation and membrane damage. Interest in this process has grown significantly over the past decade, highlighting various regulatory components. Pathways such as NF-κB and HIFs influence ferroptosis and iron metabolism, while inflammation alters iron regulatory systems, leading immune cells like macrophages and neutrophils to adopt iron-sequestering phenotypes.

Contursi et al. have reviewed the link between inflammation, platelets, and tumor progression, highlighting the potential to develop cancer prevention strategies. Platelets create an inflammatory microenvironment that supports tumor growth and metastasis. The use of antiplatelet agents, particularly low-dose aspirin, can reduce cancer risk, especially for colorectal cancer. Further research is needed on the anti-cancer effects of other antiplatelet drugs, including ADP P2Y_12_ receptor antagonists and new agents that are in clinical development, to find treatments with minimal effects on hemostasis.

Tao et al. have reviewed the multiple functions of apolipoprotein A-I (ApoA-I), a high-density lipoprotein (HDL) component. ApoA-I has a cholesterol reversal transport function and exerts anti-inflammatory effects mainly by regulating the functions of immune cells such as monocytes/macrophages, dendritic cells, neutrophils, and T lymphocytes. It also modulates the function of vascular endothelial cells and adipocytes. Additionally, ApoA-I directly exerts anti-inflammatory effects against pathogenic microorganisms or their products.

Wu et al. have examined the immunostimulatory effects of flavonoids from Epimedium, particularly icaritin and icariins I and II, both *in vitro* and *in vivo*. Key findings indicate that these flavonoids enhance the expression of co-stimulatory molecules (CD40, CD80, CD86) and MHC-I/II in dendritic cells, increasing the production of chemokines and pro-inflammatory cytokines. *In vivo*, they function as vaccine adjuvants, elevating serum levels of OVA-specific IgG. Icaritin and icariins I and II emerge as promising TLR7/8 immunomodulators with lower toxicity and higher bioavailability, potentially benefiting anticancer applications by inhibiting tumors and improving the tumor microenvironment.

Osteoarthritis (OA) is a common degenerative joint disease, and there are currently no approved treatments for modifying its progression.

Qian et al. have investigated the effects of AFK-PD, a novel pyridone agent, on OA induced by medial meniscus destabilization (DMM) *in vivo* and on chondrocytes treated with IL-1β *in vitro*. Results showed that AFK-PD reduced OA progression by inhibiting cartilage degeneration, inflammation, and osteophyte formation. It also decreased chondrocyte inflammation and macrophage M1 polarization, promoting chondrocyte anabolism and reducing catabolism and apoptosis. Mechanistically, AFK-PD suppressed key signaling molecules in the MAPK and NF-κB pathways. These findings suggest that AFK-PD could be a promising therapeutic candidate for OA treatment.

Stigmasterol, a natural plant sterol found in various herbs and vegetables with anti-inflammatory, antioxidant, and cholesterol-lowering effects, has been explored for its therapeutic potential in Acute Pancreatitis (AP) by Zhao et al. The authors utilized network pharmacology and experimental verification in a sodium taurocholate-induced AP mouse model. Analysis of protein-protein interactions revealed MAPK3 (ERK1) as a crucial target for the effects of stigmasterol in AP. Molecular docking indicated a strong binding affinity of stigmasterol for ERK1. Both *in vivo* and *in vitro* experiments demonstrated that stigmasterol treatment mitigated pancreatic injury, reduced lipase and amylase serum levels, improved inflammatory responses, and lessened acinar cell necrosis. Mechanistically, stigmasterol inhibited the activation of the ERK signaling pathway, facilitating a transition from necrosis to apoptosis in pancreatic acinar cells, thus averting inflammation.

Ammazzalorso et al. using an *in silico* approach, identified a novel chemical scaffold that is highly selective and potent in inhibiting cyclooxygenase(COX)-2 activity in inflammatory and cancer cells. AA520 is a unique molecule with dual inhibitory effects on COX-2 and PPARα at the same concentration range. Considering the synergistic impact between PPARα and COX-2 inhibitors in limiting tumorigenesis, developing molecules with these dual pharmacological targets is of clinical relevance.

Gastric contents aspiration is one of the most common causes of acute lung injury (ALI) and acute respiratory distress syndrome (ARDS), conditions associated with significant morbidity and mortality. In an HCl-induced ALI/ARDS mouse model, Dong et al. have evaluated polyvinylalcohol-carbazate (PVAC), a polymer that binds aldehydes, reducing oxidative stress and inflammation. PVAC treatment improved airway hyperresponsiveness, reduced pulmonary edema, and decreased lung damage while lowering neutrophil recruitment and inhibiting IL-6, TNF-α, and leukotriene B4 levels. These findings indicate PVAC could serve as a potential treatment for ALI/ARDS due to gastric acid aspiration and help manage asthma-like symptoms in gastroesophageal reflux patients.

Messler et al. have focused on elucidating the potential role of docosahexaenoic acid (DHA), a polyunsaturated fatty acid, in conjunction with acetylsalicylic acid (ASA) in angiogenesis, utilizing both *in vitro* and *in vivo* experimental models. The findings from these studies indicate that ASA may facilitate the formation of specific monohydroxylated metabolites of DHA, which appear to exert a significant influence over angiogenic processes.

Kiprina et al. investigated the effects of exogenous anandamide (AEA) in a mouse AirPouch model of acute inflammation through analysis of immune cell infiltrates. The study found that AEA limits the infiltration of myeloid cells but increases the presence of T cells at the site of inflammation. This action is mediated by the NR4A transcription factor instead of CB receptors. AEA effectively inhibits TH17 responses without hindering TH1 differentiation, indicating its potential for managing chronic inflammation while preserving vital immune functions.
